# A Framework for Stochastic Optimization of Parameters for Integrative Modeling of Macromolecular Assemblies

**DOI:** 10.3390/life11111183

**Published:** 2021-11-05

**Authors:** Satwik Pasani, Shruthi Viswanath

**Affiliations:** National Center for Biological Sciences, Tata Institute of Fundamental Research, Bangalore 560065, India; satwikvp@ncbs.res.in

**Keywords:** integrative modeling, molecular simulations, MCMC, stochastic sampling, derivative-free optimization

## Abstract

Integrative modeling of macromolecular assemblies requires stochastic sampling, for example, via MCMC (Markov Chain Monte Carlo), since exhaustively enumerating all structural degrees of freedom is infeasible. MCMC-based methods usually require tuning several parameters, such as the move sizes for coarse-grained beads and rigid bodies, for sampling to be efficient and accurate. Currently, these parameters are tuned manually. To automate this process, we developed a general heuristic for derivative-free, global, stochastic, parallel, multiobjective optimization, termed StOP (Stochastic Optimization of Parameters) and applied it to optimize sampling-related parameters for the Integrative Modeling Platform (IMP). Given an integrative modeling setup, list of parameters to optimize, their domains, metrics that they influence, and the target ranges of these metrics, StOP produces the optimal values of these parameters. StOP is adaptable to the available computing capacity and converges quickly, allowing for the simultaneous optimization of a large number of parameters. However, it is not efficient at high dimensions and not guaranteed to find optima in complex landscapes. We demonstrate its performance on several examples of random functions, as well as on two integrative modeling examples, showing that StOP enhances the efficiency of sampling the posterior distribution, resulting in more good-scoring models and better sampling precision.

## 1. Introduction

Integrative modeling or hybrid modeling is a method of determining macromolecular structures by combining data from complementary experiments, physical principles, statistical inference, and prior models [1,2,3]. It is used to determine structures of large macromolecular assemblies, which are refractory, to a single experimental method such as X-ray crystallography or cryoelectron microscopy [4,5,6,7,8]. First, all available information on the system is gathered. Next, a coarse-grained representation is defined for the system, and input information is translated into spatial restraints. Then, structural models consistent with the input information are sampled using a sampling procedure that is necessarily stochastic, since exhaustively enumerating all structural degrees of freedom is infeasible for large assemblies. Finally, the sampled structures are analyzed and validated [1,3,9,10,11,12,13].

Markov Chain Monte Carlo (MCMC)-based methods are used for sampling the posterior distribution of structural models, given input information [3,10,11,12,13,14]. These methods usually require the tuning of several parameters for the sampling to be efficient and accurate. Examples include Monte Carlo move sizes such as the maximum translation for coarse-grained beads, maximum translation and maximum rotation for rigid bodies, restraint-specific parameters such as restraint weights, and the temperature range for replica exchange MCMC. Monte Carlo move sizes need to be tuned such that their MCMC proposal acceptance rate is in a desired range (e.g., 0.15–0.5) [15,16]. If the move sizes are very large, most proposed moves are not accepted, which renders the sampling inefficient. On the other hand, if the move sizes are very small, a large number of steps are required to converge to the stationary distribution, which again renders the sampling inefficient. The optimal scaling of move sizes ensures efficient convergence to the stationary distribution, i.e., efficient sampling [15,16]. To put it another way, if the available computing time is limited, optimal scaling of move sizes ensures that the structures sampled in the given time more accurately resemble the posterior distribution.

Novice modelers are not aware of how to tune these parameters in integrative modeling. In the best case, currently, these parameters are tuned manually, a procedure that is both error prone and time consuming, since it requires a starting point, step size, and gradient, usually only allows tuning one parameter at a time, and ignores the dependency between parameters. Moreover, manual tuning is infeasible for complex landscapes with lots of dependent parameters. Here, we introduce a heuristic to replace this manual procedure.

Broadly, the problem is of finding solutions (values) for the sampling-related parameters (e.g., MCMC move sizes) such that their corresponding metrics (e.g., MCMC proposal acceptance rate) are in the target range. These metrics can be considered as objective functions that are optimized over the search space of parameters. In a single modeling setup, there can be several metrics. Each parameter can affect multiple metrics, and each metric can depend on multiple parameters. This corresponds to a multiobjective optimization problem, with each objective function corresponding to a high-dimensional, complex landscape whose functional form is not known analytically. Moreover, the optimization problem is stochastic since the objective functions are evaluated via sample MCMC runs.

One can use adaptive jump proposals to automate the tuning of MCMC parameters. However, these proposals are not Markovian; the convergence to stationary distribution cannot be easily verified in all cases [17]. Moreover, they require extensive reimplementation in the Integrative Modeling Platform (IMP) [13], leading us to consider alternative approaches. In the field of molecular dynamics, sampling-related parameters such as force-field parameters are sometimes optimized by gradient descent [18]. In our case, we do not know the functional form of our metrics. Hence, we rely on derivative-free optimization methods, i.e., optimization methods that do not rely on gradients [19].

We mention related derivative-free optimization methods here and contrast specific properties of these methods with ours in Section 4. First, local, deterministic optimization methods include the Nelder–Mead simplex method and pattern-search methods. In the Nelder–Mead simplex method, for an *n*-dimensional search, a simplex is created with n+1 candidate points as its vertices. At each iteration, the vertices of the simplex are modified based on their objective function values [20]. In pattern-search methods, on the other hand, at each iteration, a finite set of candidate points $x_{k+1}=x_{k}+a_{k}d$ are generated near the current point, $x_{k}$, with methods differing in how the step sizes $a_{k}$ and directions d are chosen [21,22,23,24,25,26]. Several of these methods are univariate methods, i.e., they optimize over *n* dimensions by searching over one dimension at a time, keeping the remaining n−1 dimensions fixed.

Second, local, stochastic optimization methods include methods where the objective function is stochastic, such as stochastic approximation (SA), and methods where the candidate points are generated at random, such as random search-based methods. SA methods optimize stochastic objective functions by iteratively estimating their gradients via finite differences. They however, require that the objective functions are strongly concave or convex [27,28]. Random search-based methods, such Rastrigin’s random search and recent variates, iteratively sample a random position in an *n*-dimensional hypersphere centered at the current point [29].

Third, global, deterministic optimization methods can be one-dimensional or multidimensional searches. One-dimensional searches such as binary search [30], interpolation search [31], and golden-section search [32] successively narrow down the search space by evaluating the objective function at one or two new candidate points per iteration. Multidimensional searches include grid search, Shubert’s method [33], DIRECT method [34], and multilevel coordinate search (MCS) [35]. In grid search, function values are evaluated at all points in an *n*-dimensional grid, whereas, in Shubert’s method, DIRECT, and MCS, the search space is partitioned into hyper-rectangles and progressively narrowed down at each iteration based on function values at points inside or at the corners of these hyper-rectangles [33,34,35].

Finally, global stochastic optimization methods include pure random search (PRS), where candidate points are generated at random from the search space [19], evolutionary population-based methods, such as genetic algorithms [36] and particle swarm optimization [37], and Bayesian optimization where the unknown objective function is treated as a random function modeled by a prior [38]. Our method is similar to DIRECT and MCS in progressively narrowing down the search space into smaller spaces along a single-coordinate axis. However, a direct application of these methods is not possible since we require our objective functions to lie in a given range instead of being maximized or minimized; our problem is stochastic, and it involves multiple-objective functions.

Here, we develop a general heuristic for derivative-free, global, stochastic, parallel, multiobjective optimization, termed StOP (*Stochastic Optimization of Parameters*), that we apply to optimize sampling-related parameters for integrative modeling. The method makes flexible use of the available computing capacity and facilitates the optimization of several parameters at a time. The only assumption about the metrics, i.e., objective functions, is that they are continuous functions. On the other hand, StOP requires the user to know the parameter–metric mapping for the specific system being modeled, is not efficient for very high-dimensional landscapes, and is not guaranteed to find solutions in a complex landscape. We demonstrate the method on example functions as well as on real integrative modeling cases. StOP improves the efficiency of sampling the posterior distribution, which in turn enhances the number of good-scoring models and the sampling precision [9].

## 2. Methods and Materials

### 2.1. Theory and the Algorithm

In this section, we begin with an example problem, introduce the notation, and then develop the theory underlying StOP, starting with basic search strategies. We then present the final StOP algorithm and explain the practical implementation details.

#### 2.1.1. Example

For the illustration of the method we begin with the context of a protein–protein docking problem simulated using Integrative Modeling Platform (IMP) [13]. We call the proteins A and B. For both A and B, the regions with known tertiary structure are represented by rigid bodies, while the remaining regions are represented by flexible beads. We consider three parameters (move sizes) that need to be optimized in this modeling setup, the *rigid-body-maximum-rotation* (RBRot), *rigid-body-maximum-translation* (RBTrans), and the *flexible-bead-maximum-translation* (FBTrans). Our goal is to have the average Monte Carlo acceptance probabilities corresponding to the above move sizes lie in a given target range, which we choose to be [0.3,0.6]. These probabilities are the metrics to be optimized, represented by *Protein-A-rigid-body-MonteCarlo-acceptance-ratio* (RB-A), *Protein-B-rigid-body-MonteCarlo-acceptance-ratio* (RB-B), and *flexible-beads-AB-MonteCarlo-Acceptance-Ratio* (FB-AB), with the target range of each of these metrics being [0.3,0.6]. We choose to separately optimize the MC acceptances of the rigid bodies in proteins A and B, while the MC acceptances of flexible beads in both the proteins are optimized together, to minimize the number of optimized parameters and the metrics.

#### 2.1.2. Notation

The input of our optimization problem is defined by *n* parameters of a modeling setup which require optimization, represented by the vector $x=\left(x_{1},x_{2},\cdots ,x_{n}\right)\in R^{n}$. For each input parameter $x_{i}\in R$, we define the *input domain* of this parameter, i.e., this parameter’s search space as $[c_{i},d_{i}]\subset R$. In our example with n=3, we have x=(RBRot,RBTrans,FBTrans). We further have a set of *k* functions of the parameters $\left\{F\right\}=\left\{f_{1}\left(x\right),f_{2}\left(x\right),\cdots ,f_{k}\left(x\right)\right\},f_{i}:R^{n}\to R$, which represent the metrics of our modeling setup and are the objective functions for our optimization problem. For each metric, we also set an optimal target range as $[a_{i},b_{i}]\subset R$. In our example, $\left(f_{1},f_{2},f_{3}\right)=\left(\mathrm{RB}-A,\;\mathrm{RB}-B,\;\mathrm{FB}-\mathrm{AB}\right)$.

The goal of the optimization algorithm is to find $x^{*}$, such that for each $f_{i}\in \left\{F\right\}$ we have $f_{i}\left(x^{*}\right)\in \left[a_{i},b_{i}\right]$. The optimization algorithm at each step has a collection of *candidate points* which are parameter values that are being explored as possible solutions $x^{*}$. At each stage of the optimization, the search space of the parameter that is being explored and may possibly contain some solutions is called a *feasible range*. The feasible range at the beginning of optimization is the input domain of the parameter. Finally, the range of values of x, for which a given metric lies in the target range, is referred to as a *desired range* of x for that metric.

All these metrics are calculated on the results of MCMC sampling and, hence, are inherently stochastic. To minimize this stochastic variation, the metrics used for optimization are the expected values of these stochastic underlying functions, approximated by averaging over a prespecified number of replicates, i.e., repeated IMP runs with the same x as the input. The metric function used in optimization, $f_{i}$, is related to the noisy underlying function calculated on the results of a single run, $f_{i}^{\prime }\left(x,\eta \right)$, where η is some random variable representing stochastic *noise* as $f_{i}\left(x\right)=E_{\eta }\left[f_{i}^{\prime }\left(x,\eta \right)\right]$.

#### 2.1.3. Partitioning into Groups

**Figure 1:**
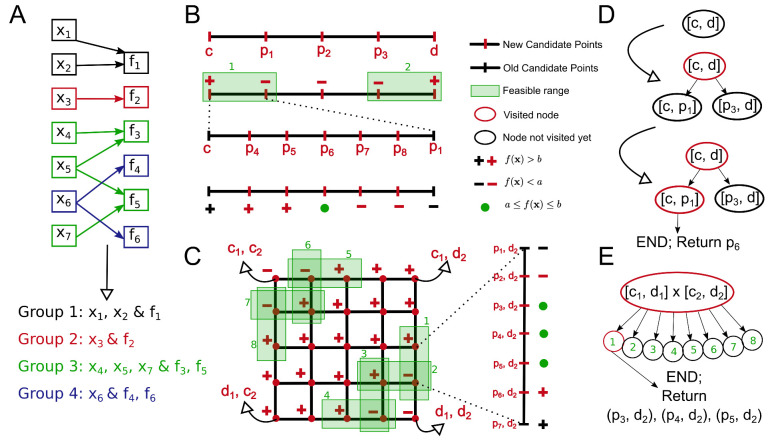
Illustrations of the basic concepts of StOP. (**A**) Partitioning of the input parameters and the metrics into disjoint groups based on the parameter–metric graph. Different groups are shown in different colors. (**B**) *m*-ary search for a one parameter, one metric example with m=5,n=1. The metric is represented by f(x), the target range of the metric by [a,b], and the input domain of the parameter by [c,d], $p_{i}$ represents candidate points at different steps. “+” and “–” represent the metric values greater than and lesser than the target range, respectively. Starting from the input domain, we find two feasible ranges $\left[c,p_{1}\right]$, $\left[p_{3},d\right]$ (numbered and highlighted in green). In the next step, we explore one of them, $\left[c,p_{1}\right]$, and we find a solution $p_{6}$. (**C**) 2-D-*m*-ary search for a two parameter, one metric example with m(2)=m(1)=5, n=2. The input domains of the two parameters are $\left[c_{1},d_{1}\right]$ and $\left[c_{2},d_{2}\right]$, respectively. We explore candidate points in the first step, finding 8 feasible ranges (numbered and highlighted in green). In the second step, we fix the value of the second parameter at $d_{2}$, searching along the first feasible range producing three solutions $\left(p_{3},d_{2}\right),\left(p_{4},d_{2}\right),\left(p_{5},d_{2}\right)$. (**D**) The evolution of the DFS tree for the example in (**B**). (**E**) The final DFS tree for the example in (**C**). The legend is common for (**B**–**E**).

Each metric may not be affected by all parameters. In our example, we might assume that the rigid-body parameters do not affect the flexible-bead MCMC acceptance rate and vice versa. This allows us to separate our parameters and metrics into groups such that each group contains only the parameters which have an effect on the corresponding set of metrics in the group and do not affect the metrics of the other group. Formally, the metrics in a given group are functions of only the parameters in that group. This knowledge can be captured by an input bipartite graph from the set of parameters to the set of metrics where a link $x_{i}\to f_{j}$ signifies that $f_{j}$ is a function of $x_{i}$. Given such a graph, we can divide x and {F} into disjoint groups, where each group is a maximally connected subgraph (Figure 1A). We obtain *g* such groups, and the set of parameters in the *i*-th group is represented by the set $A_{i}^{x}$ and the set of metrics in the *i*-th group by the set $A_{i}^{f}$. It follows from this that $|A_{i}^{x}|=n_{i}$, $\sum n_{i}=n$, $|A_{i}^{f}|=k_{i}$ and $\sum k_{i}=k$ with $n_{i}$ and $k_{i}$ being the number of parameters and metrics in group *i*, respectively.

For each group *i*, we consider each $f_{j}\in A_{i}^{f}$ to only be a function of the parameters in $A_{i}^{x}$, i.e., $f_{j}\left(A_{i}^{x}\right):R^{n_{i}}\to R$, and it does not depend on the remaining parameters. The notation used here is $f\left(\left\{x\right\}\right):=f\left(x_{1},x_{2},\cdots ,x_{n}\right)$ with $\left\{x\right\}=\left\{x_{1},x_{2},\cdots ,x_{n}\right\}$ for any arbitrary ordering. The metrics and parameters of a group are independent from those of any other group and can be optimized without considering the values, state, or any other information of the optimization progress of the other groups.

Using the formal notation for our example, we have g=2 and $A_{1}^{f}=\left\{\mathrm{RB}-A,\;\mathrm{RB}-B\right\}$ and $A_{2}^{f}=\left\{\mathrm{FB}-\mathrm{AB}\right\}$. For the parameters, we have $A_{1}^{x}=\left\{\mathrm{RBRot},\;\mathrm{RBTrans}\right\}$ and $A_{2}^{x}=\left\{\mathrm{FBTrans}\right\}$. For now, only consider group 2 with a single parameter (*x*) and a single metric (*f*), and we discuss the more general case later.

#### 2.1.4. Basic Optimization Strategies: Manual, Binary, *m*-ary

##### 2.1.4.1. Manual Search

The manual search strategy begins with a modeler-specified starting value of the parameter. This is followed by IMP MCMC sampling with that parameter value, analyzing the sampling result, and deciding how to change the parameter value to push the metric toward the target range. This requires some knowledge of the gradient of the metric with respect to the parameter. Usually, the modeler only needs to know the sign of the gradient. Assuming the parameter–metric relation to be monotonic, the modeler makes steps of a given size in the direction that is likely to push the parameter toward the desired range.

For example, assume that the FBTrans to FB-AB landscape is monotonic and that increasing FBTrans decreases FB-AB. If at the arbitrarily chosen initial value of FBTrans, e.g., 4 Å, we obtain the value of FB-AB as 0.9, which is higher than our target range of [0.3,0.6], we would increase the FBTrans by a step size of 2 Å, to, e.g., 6 Å.

##### 2.1.4.2. Binary Search

Automating the above process while requiring similar information from the modeler, and translating it into a faster-converging algorithm, we can update the parameter by performing a 1-D binary search of the parameter input domain using the same assumed monotonic parameter–metric relationship without the need of the step size or the initial point. We begin in the center of a range assumed to contain the desired range of *x*. Based on the assumed sign of the gradient function and the fact that the metric for the central parameter value overshot or undershot the target range, we can reject half the range of *x* for the next iteration. We repeat the process again on the remaining search space. We halve the search space of the parameter while exploring one new candidate point at each step.

##### 2.1.4.3. *m*-ary Search

To remove the need to know the correct sign of the gradient function and to allow for an algorithm more robust to non-monotonic landscapes, we can then employ an *m*-ary search where m≥2. In practice, *m* can be much larger than 2. Instead of sampling just the midpoint, we sample *m* linearly spaced points along the parameter feasible range at each iteration. This assumes the x→f map, for our case, the FBTrans to FB-AB map, to be continuous. Given a continuous map, if the metric value at two of the adjacent candidate points flank the target range of *f*, by the intermediate value theorem, the metric function values between those two points must contain the target range. The range bound by the two candidate points can then become the feasible range for the next iteration. Figure 1B illustrates an example *m*-ary optimization with m=5.

For monotonic functions, *m*-ary search always finds this range given a sufficiently large input domain for *x* such that it contains the desired range of *x*. This works faster than 1-D binary search by rejecting (m−2)/(m−1) of the parameter search space in the first iteration and m/(m+1) at each successive iteration, with *m* new candidate points explored per iteration utilizing the parallel computing capacity better.

#### 2.1.5. The General Algorithm

We can now look at the general case of multiple nonsingleton groups of parameters and metrics. The metrics of each group can be optimized independently (see Section 2.1.3).

##### 2.1.5.1. *n*-D Search and DFS

For now, assume that a given group *i* has $n_{i}$ parameters and a single metric. We employ at the first iteration an $n_{i}$-D-*m*-ary grid search where we sample the metric across a $n_{i}$-dimensional grid instead of a 1-D line where each axis of the grid represents one parameter that changes along the axis while all other parameters remain fixed, i.e., sample all $m^{n_{i}}$ combinations of the *m* linearly spaced points, initialized within the parameter input domain, along $n_{i}$ axes (Figure 1C). We then explore all the candidate points in this grid. A feasible range is found if the metric values at two adjacent grid points (non-diagonal) flank the target range. These ranges lie along a grid line; hence, all the subsequent searches are 1-D-*m*-ary searches, where we optimize only one parameter with the remaining parameters fixed at a particular value.

As a global optimization strategy, we employ Depth First Search (DFS), a type of branch-and-bound search used in several global optimization problems. Each node in the DFS tree represents a feasible range. For any given node, the children nodes are created after exploring all the candidate points at the node and they represent the feasible ranges found from the given node. Hence, the root node of all the groups consists of a $n_{i}$-D search node, and all other nodes consist of 1-D search nodes. To continue DFS, one of these children nodes is selected for exploration. A node either ends in success (a solution point found) or failure which can be due to the failure to find any feasible ranges (no children nodes) or due to exceeding the maximum allowable depth of the DFS tree (see Section 2.1.5.2). In case of failure, we mark the node as visited and continue DFS without creating any children nodes. The overall search ends when all nodes are visited or a solution point is found at any node. Figure 1D,E illustrates the DFS tree for the examples in Figure 1B,C, respectively.

For a nonstochastic metric continuous in all the input parameters, the multivariate extension of the intermediate value theorem guarantees that given a feasible range as defined above, there must be a desired range within this feasible range. This is an expensive strategy that works well for small $m,n_{i}$ but can easily become impractical at high values for *m* and $n_{i}$.

##### 2.1.5.2. Maximum DFS Depth and DFS versus BFS

We fix the maximum depth of the DFS tree, which curtails the maximum running time of StOP. Apart from practical reasons, this also helps in cases where the stochasticity of the metrics causes a subrange to be falsely classified as a feasible range. In this case, the DFS may indefinitely continue down this misclassified branch, unless the maximum depth is fixed.

Since only a single solution is required, DFS is preferable over Breadth First Search (BFS) as the latter explores all the nodes at a given depth before moving on to a higher depth, while the former fully explores a feasible range before backtracking. In addition, BFS might greatly increase the average runtime in case of landscapes where the desired range is small in comparison to the input domain. Although BFS would be more robust to the above issue of misclassified branches, chances of such a stochastic misclassification are low if the target range is wide and if the number of replicates is reasonably high.

##### 2.1.5.3. Multiple Metrics in a Group

**Figure 2:**
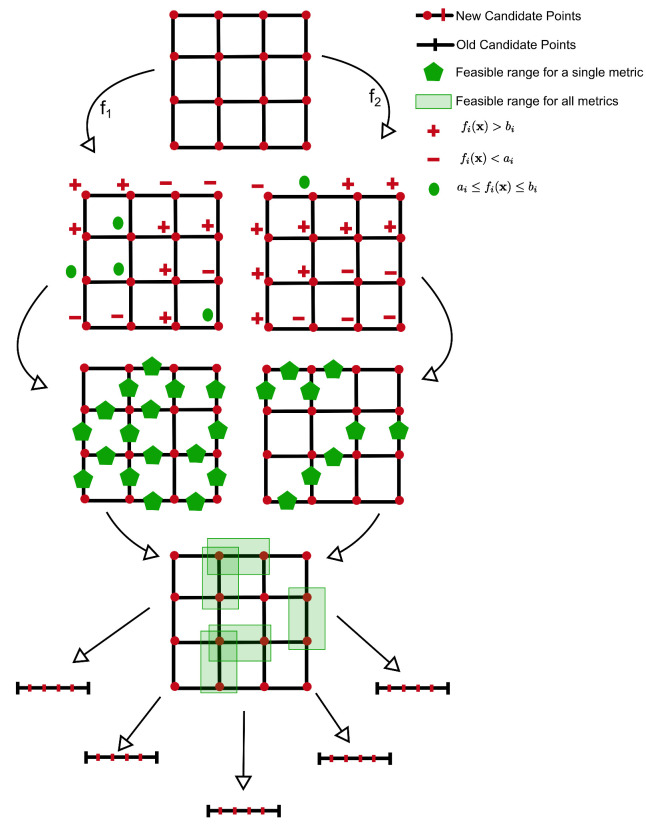
Illustration of StOP for a multimetric example. 2-D-*m*-ary search for a 2-parameter 2-metric example where the two functions are denoted by $f_{1}$ and $f_{2}$ with the corresponding target ranges as $\left[a_{1},b_{1}\right]$ and $\left[a_{2},b_{2}\right]$. Metric-specific feasible ranges are calculated similar to the single-metric case but only the feasible ranges common for both the metrics are explored further. m=4,n=2 for this example.

Finally, the metric set $A_{i}^{f}$ may have more elements, as is the case in our example for group 1. To modify the above search strategy to allow for this, we calculated the feasible ranges separately for each metric. For a multimetric problem, we considered as a feasible range any range that is a feasible range according to all of the metrics in the set $A_{i}^{f}$. Figure 2 shows a multimetric optimization example of a group with 2 parameters and 2 metrics.

Unlike the previous case with a singleton set $|A_{i}^{f}|=1$, finding a feasible range in a node does not guarantee that there exists a region in that range where all the metrics are satisfied since different metrics may have nonoverlapping desired ranges in this range. Backtracking along the failed branches of the DFS tree allows us to continue a global exploration in case a given node failed due to the absence of feasible ranges in case of nonoverlapping desired ranges.

#### 2.1.6. StOP: Algorithm and Practical Enhancements

The final algorithm follows the strategy as described above with some practical enhancements.

##### 2.1.6.1. General Flow

**Figure 3:**
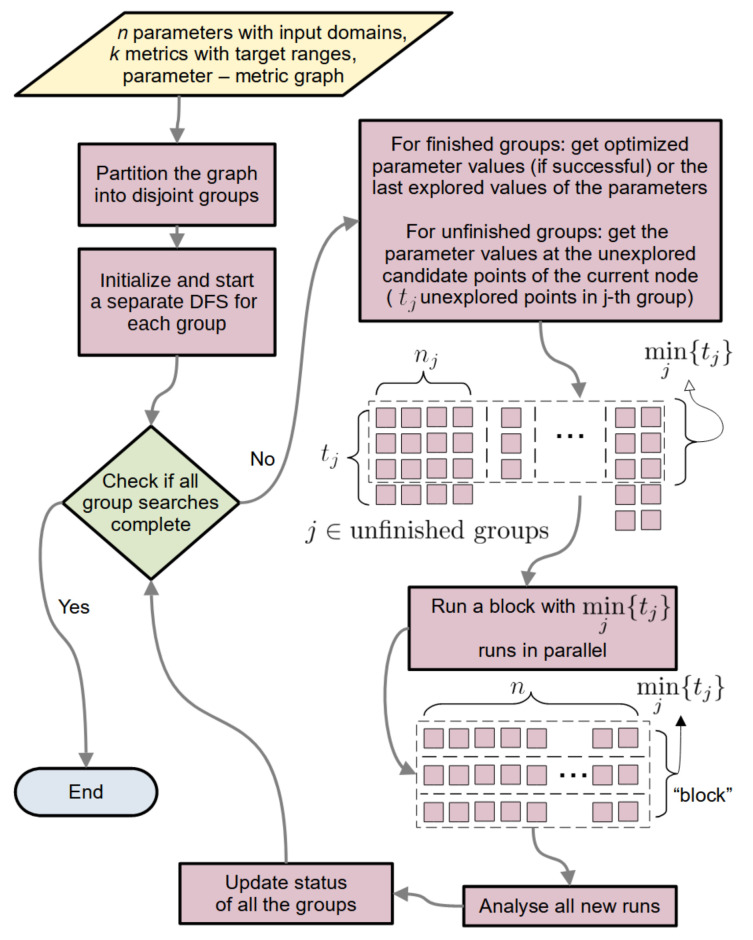
Flowchart for the core algorithm of StOP.

Figure 3 outlines the general StOP algorithm. The modeler inputs three things: the list of parameters to be optimized and the metrics affected by each parameter (Figure 1A), the target range for each of the metrics and the input domains for the parameters (Section 2.1.2), and the optional StOP-specific parameters including the maximum allowed depth of the DFS tree, the value for *m* (see Section 2.1.6.4), number of replicates per candidate point, and other analysis parameters specific to IMP (see Supplementary Section S1).

The StOP algorithm begins with the parameter groups and optimizes them simultaneously until all the group searches are complete, either successfully, i.e., a solution point found, or unsuccessfully, i.e., the DFS ended without any solution points. At a given step, the node currently being explored (current node) in the DFS tree of group *j* may have $t_{j}$ unexplored candidate points in the search grid of the node. Since every IMP run requires us to specify all the parameters, only a limited number of IMP runs can be parallelized. This set of runs is called a block here, and at any step comprises $\min_{j}\left\{t_{j}\right\}$ runs. Groups with the search completed are removed from this calculation. Parameters in these groups are fixed to either a solution point if the group search ended successfully or to the last candidate point explored. After all the runs in a parallel block finish, they are analyzed, and the group states are updated as outlined below.

##### 2.1.6.2. Updating the Group State

**Figure 4:**
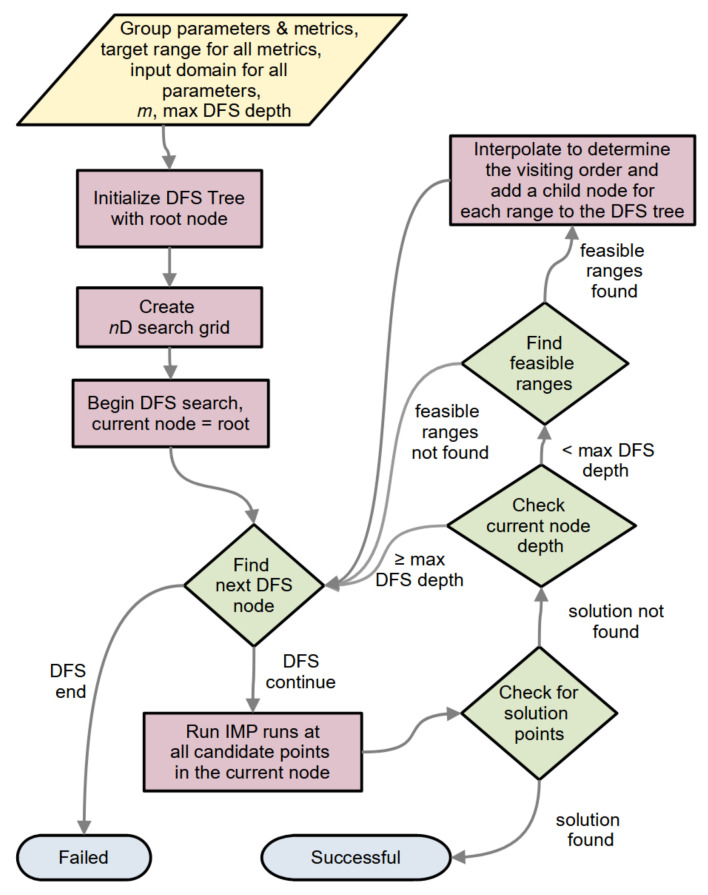
Flowchart of StOP for a single group.

Figure 4 outlines the StOP algorithm for a single group and describes the process of updating the group state in the general flowchart in Figure 3. If some of the candidate points in the current node of a given group *j* are not explored, StOP simply updates the value of $t_{j}$ and awaits the completion of the remaining runs. If all the points are explored, StOP proceeds to find feasible ranges or solution points in the current node, creating a child node from each discovered feasible range, and updating the group state to completed if a solution point is found or if the DFS search has ended.

##### 2.1.6.3. Setting the Visiting Order for the Nodes

If multiple feasible ranges are discovered in a node, the order of adding these children nodes to the tree and the subsequent relative order of traversal should involve some measure of the probability of finding a solution point in these ranges. This, in turn, depends upon the size of the desired range within the feasible range. We use an interpolation-based heuristic to guide this order. The metric values at the candidate points are interpolated using a cubic or a quadratic spline, and the interpolated function is evaluated at 100 linearly spaced parameter values within each of the feasible ranges. The ranges are prioritized in a descending order of the proportion of these queried points lying in the metric target range for all metrics.

##### 2.1.6.4. CPU Economy and m(n)

Since an *n*-D search becomes exponentially expensive with *n*, setting the same value of *m* for all *n* makes either the low-dimensional searches inefficient or makes the higher-dimensional searches impractical. To allow for this, StOP allows specifying *m* as a function of *n*, m(n), to balance the tradeoff between the runtime and optimization robustness. Further, the parallel block size at the different steps depends on the number of candidate points in the different groups. To optimize the utilization of the maximum number of parallel processes on the machine, one of the recommended specification for m(n) is as follows: based on the $n_{i}$ for the different groups in the given optimization problem, the value $m{\left(n_{i}\right)}^{n_{i}}$ for all the $n_{i}$ should be a multiple of m(1), which ensures that the blocks at least contain m(1) runs at all steps. In case it is not a multiple, some blocks contain less than m(1) runs, which may result in a suboptimal utilization of the available parallel computing capacity.

Furthermore, the number of maximum available processes should be a multiple of m(1) such that the number of replicates is equal to or a multiple of the quotient, which allows all the available processes to always be used. For example, on a machine with 8 maximum processes available, we can set m(1)=8,m(2)=4,m(3)=4, and any number of replicates. By default, StOP uses a strategy that attempts to satisfy these restraints while choosing the default m(n) (see Supplementary Section S2), but it is recommended for the user to adapt m(n) to their use case.

### 2.2. Illustrative Examples

#### 2.2.1. Example Functions

We demonstrate StOP on four example cases where we add Gaussian noise to arbitrarily chosen polynomial functions. StOP is evaluated on a more thorough unbiased set of examples with a random selection of landscapes in Supplementary Section S3. This also includes a comparison of different values of *m* and their effect on the success of StOP on more complicated landscapes.

#### 2.2.2. Comparison to Genetic Algorithm

We compared StOP to a Genetic Algorithm (GA) search on 20 random 2D functions (Supplementary Section S3, h=5). The GA-search had a population size, i.e., number of candidate points per generation, of 5 and 50. The former matches the value of m(n) for StOP resulting in a fairer comparison. The fitness of any candidate point was defined as the negative of the absolute distance of the metric value at that candidate point and the closest edge of the target range. In each iteration of the GA-search, 25% of the fittest candidate points were carried forward (retained) to the next generation. Mutation operation included adding a random value to the candidate points, which was generated as a random number uniformly distributed between ± maximum mutation size. The mutated candidate point was, however, constrained to lie in the input domains of the parameters. In total, 75% of the population of the next generation was generated by mutating the retained population, and the remaining 25% was the unaltered retained population. Finally, a crossover step was applied to this population where a randomly chosen subset half the size of the population is selected. Random pairs from this subset were crossed over. We ran the GA-search 10 times for each metric resulting in 200 total searches to mitigate the effect of random initializations. We repeated this for different values of the maximum mutation size ranging from 0.02 to 0.5, which corresponds to 1–25% of the size of the input domain. To curtail the run time, we allowed a maximum of 10,000 metric evaluations, which corresponds to 2000 and 200 generations for the populations sizes 5 and 50, respectively. We also evaluated StOP with m(2)=m(1)=5 on these 20 landscapes for comparison.

#### 2.2.3. Integrative Modeling Examples

We used two example systems to illustrate the difference in the sampling efficiency and exhaustiveness resulting from using optimized and unoptimized parameters: actin–tropomyosin complex [11] and the γtusc–Spc110 complex [39]. The actin system is a three subunit complex composed of actin, gelsolin, and tropomyosin. Its integrative structure was modeled with Bayesian EM (electron microscopy), Bayesian chemical crosslinking, SAXS (small angle X-ray scattering), connectivity, and excluded volume restraints. The γtusc system is a five subunit complex composed of Spc97, Spc98, Tub4 dimer, and Spc110. Its integrative structure was modeled with Bayesian chemical crosslinking, connectivity, and excluded volume restraints.

For both the systems, we first optimized the MCMC parameters using StOP. Then, we compared three types of sampling: (a) sampling with optimized parameters (*optimal*), (b) sampling with parameters 1–2 orders of magnitude greater than the optimal values (*high*), and (c) sampling with parameters 1–2 orders of magnitude lower than the optimal values (*low*). The number of sampled models was the same in all three cases. For the actin–tropomyosin system, we additionally implemented two time-controlled runs where the sampling was performed as for the *high* and *low* sets described above for the same time that the *optimal* set takes on average (*timed-high* and *timed-low*). For the γtusc–Spc110 system, we implemented two more types of sampling, with parameters 10 times higher (*med-high*) and with parameters 10 times lower (*med-low*) than the optimized parameter values, compared to 100 times higher or lower in the *high* and *low* set, respectively. The analysis pipeline used is the same as from [9,11,39].

The parameters optimized for the actin system included the flexible bead maximum translation (*optimal*: 4, *high/timed-high*: 20, *low/low-timed*: 0.001), tropomyosin rigid body maximum translation (*optimal*: 0.4, *high/timed-high*: 10, *low/low-timed*: 0.001) and maximum rotation (*optimal*: 0.1, *high/timed-high*: 2, *low/low-timed*: 0.001), actin-gelsolin rigid body maximum translation (*optimal*: 0.1, *high/timed-high*: 10, *low/low-timed*: 0.001) and maximum rotation (*optimal*: 0.1, *high/timed-high*: 2, *low/low-timed*: 0.001). In total, 20 replicates were run without replica exchange for all the sets, and the number of frames for the untimed sets was 10,000. To obtain good-scoring models, the sampled models were filtered based on 100% crosslinking data satisfaction at a distance threshold of 30 Å. A second filtering using the other restraints was applied based on a threshold of 0.25 SD below the mean of the models satisfying the crosslinking criteria for the optimal set.

The parameters optimized for the γtusc system include the γtusc maximum translation (fixed at 12.8 for all runs) and Spc110 maximum translation (*optimal*: 6.9, *med-high*: 70, *med-low*: 0.7, *high*: 700, *low*: 0.07). In total, 30 replicates with 8000 frames each were run without replica exchange for all the sets. To obtain good-scoring models, the sampled models were filtered based on 60% crosslinking data satisfaction for EDC and 50% for DSS cross-links at distance thresholds of 25 Å and 35 Å, respectively. A second filtering using the other restraints was applied based on a threshold of 0.25 SD below the mean of the models satisfying the crosslinking criteria for the optimal set.

Details of the additional software packages can be found in Supplementary Section S4 [40,41].

## 3. Results

**Figure 5:**
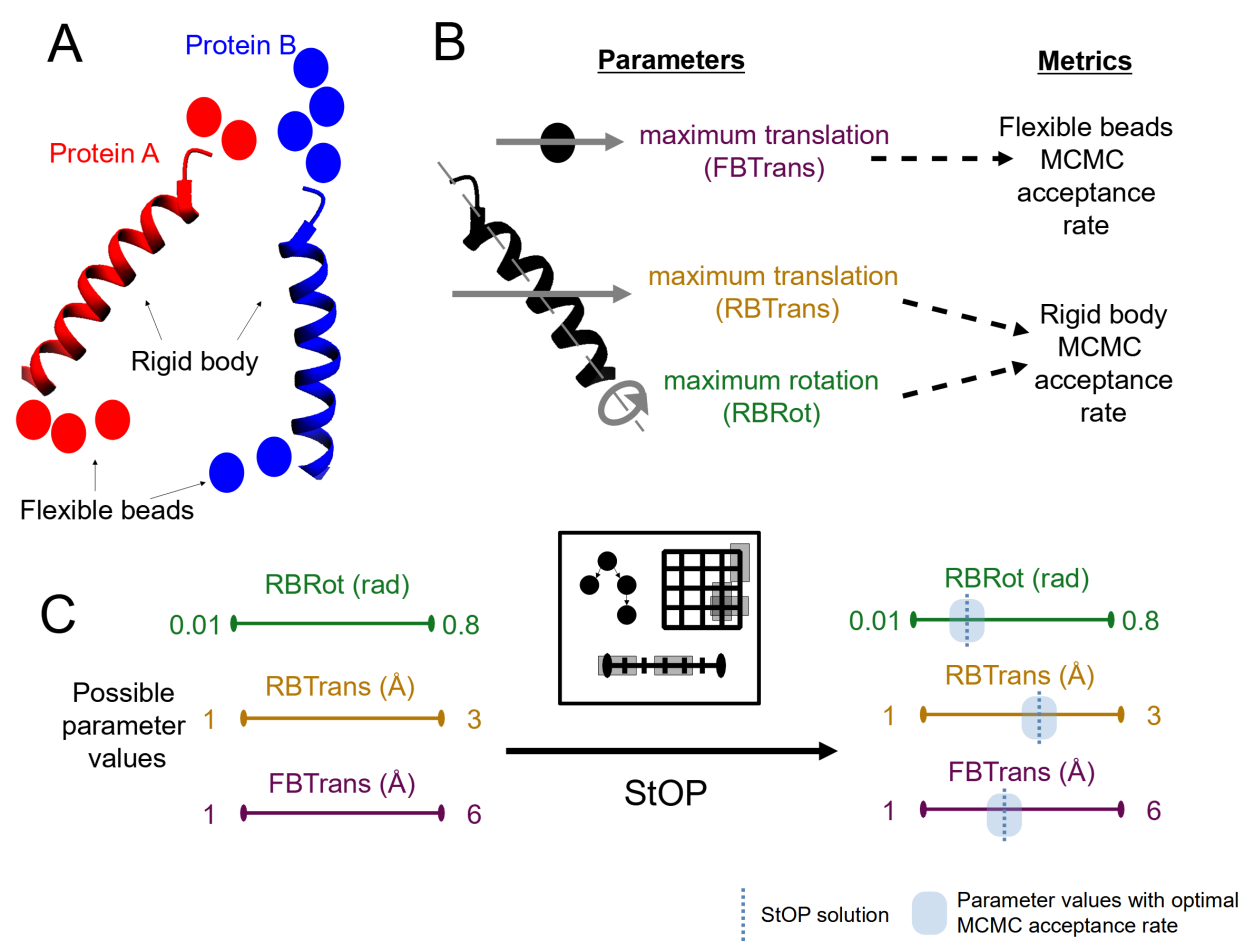
Schematic demonstrating the StOP workflow. (**A**) Integrative modeling of a protein–protein complex formed by proteins A and B. The sampling degrees of freedom include a rigid body and 4–6 flexible beads for each protein (**B**). The MCMC parameters related to move sizes for each degree of freedom (left) and the corresponding metrics, i.e., MCMC acceptance rates that they influence (right) are shown. (**C**) The parameters can take a range of values (the input domains). StOP optimizes the values of these parameters for optimal MCMC acceptance, resulting in efficient sampling.

Figure 5 depicts the overall schematic for StOP. In a typical Integrative Modeling problem, there are several proteins, each of which is comprised of one or more rigid bodies representing regions of known structure, interleaved with flexible beads representing regions of unknown structure. The positions and the orientations of the rigid bodies and the positions of the flexible beads are sampled stochastically via MCMC (Markov Chain Monte Carlo) methods to determine the integrative structure of the assembly. The corresponding MCMC parameters are the move sizes, i.e., the maximum translations and rotations for rigid bodies and maximum translations for flexible beads. These parameters are optimized by StOP such that their MCMC acceptance rates are in the target range.

We first demonstrated the performance of StOP on example functions. We then theoretically compared its performance to binary search and Genetic Algorithm search. We finally showed two example macromolecular systems simulated using IMP and compared the efficiency of sampling with optimized and with unoptimized MC parameters to illustrate the utility of StOP in real IMP systems.

### 3.1. StOP Applied to Example Functions

**Figure 6:**
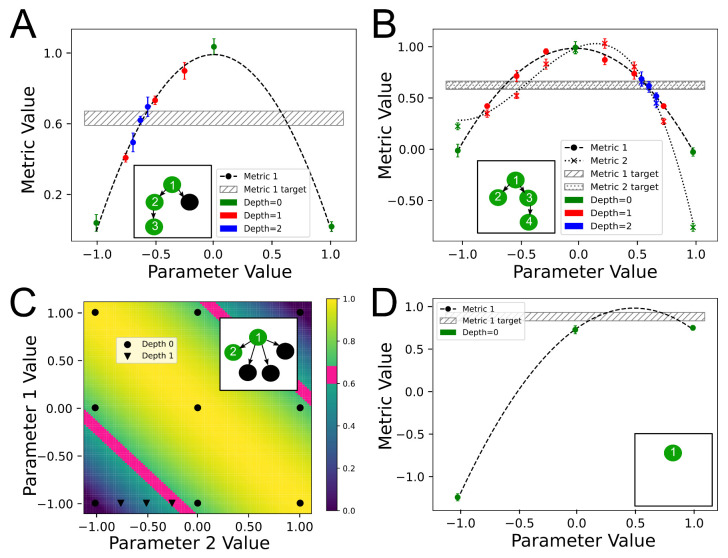
StOP applied to example functions. (**A**) A successful one-parameter one-metric search with the candidate points at different depths shown in different colors. The metric function is $1-x^{2}$ with the target range shown as the shaded region ([0.6,0.68]). Inset corresponds to the final DFS tree with green nodes representing nodes that were visited while the black nodes were not. The nodes are numbered in the order of their visit during optimization. (**B**) A successful one-parameter two-metric search with the inset showing the DFS tree with the same coloring and numbering scheme as in A. The function used for metric 1 is $1-x^{2}$ and for metric 2 is $1-x^{3}-1.2x^{2}+0.5x$ with the target range of both the metrics being fixed to [0.6,0.68]. (**C**) A successful two-parameter one-metric example with the DFS tree as inset. The metric function used is $1-{\left(\left(x_{1}+x_{2}\right)/2\right)}^{2}$, and the background colors represent the metric function landscape with pink representing the target range ([0.6,0.68]). (**D**) An unsuccessful one-parameter one-metric search with the DFS tree as inset. The metric function is $1-{\left(x-0.5\right)}^{2}$ with the target range as [0.85,0.95]. For all the panels, stochastic noise added to the metrics is Gaussian with mean 0 and standard deviation 0.05. m(1)=3 for all the panels. m(2)=3 for (**C**). The input domain of all the parameters is [−1,1].

Figure 6A shows a one-parameter one-metric system where StOP successfully manages to find a solution at depth 2 with m(1)=3. The green markers demonstrate the candidate points explored in the root node at depth 0, with two identified feasible ranges at this level, [−1,0] and [0,1], of which the former is chosen by StOP for exploration at depth 1. The red points represent the candidate points at depth 1 which identify a single feasible range. This is further explored by the blue candidate points at depth 2 where a solution is found.

For a more complicated example, Figure 6B shows a one-parameter two-metric system where StOP successfully finds a solution at depth 2 with m(1)=3. However, this also shows an example of a feasible range ([−1,0]) discovered at depth 0 according to both the metrics. However, on exploration at depth 1, no further feasible ranges or solution points are found inside this range. This is because the metric-specific feasible ranges at depth 1 for metric 1 ([−0.25,−0.5]) and metric 2 ([−0.5,−0.75]) do not overlap. This requires StOP to continue DFS and explore a sibling node at depth 1.

Figure 6C shows a successfully optimized two-parameter one-metric system where the nine round markers show the candidate points at the root node with m(2)=m(1)=3. Four feasible ranges are found, [(−1,−1),(0,−1)], [(−1,−1),(−1,0)], [(1,1),(0,1)], [(1,1),(1,0)] of which the first is explored at depth 1.

Finally, Figure 6D shows an example where StOP fails to find any feasible range and results in an unsuccessful optimization attempt with m(1)=3. This could be improved by increasing the m(1) or by altering the input domain of the parameter. The performance of StOP on a set of randomly chosen landscapes is shown in Supplementary Section S3.

### 3.2. Comparison of StOP to Binary Search

**Figure 7:**
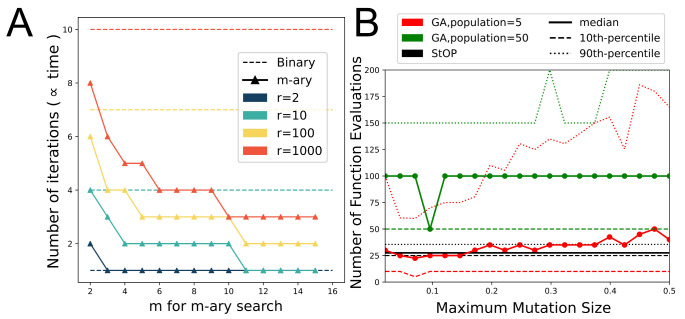
Comparison of binary search and genetic algorithm search with StOP. (**A**) A comparison of the maximum number of iterations needed to optimize a single, continuous, monotonic metric for binary versus *m*-ary search for different *m* and different values of *r*, the ratio of the parameter input domain and the desired range. The equation for the number of iterations, calculated based on the reduction in the search space per iteration, for *m*-ary search is $\left\lceil \log(r/(m-1))/\log(m+1)+1\right\rceil$. (**B**) A comparison of the number of metric function evaluations required for convergence for StOP and for GA search for 20 randomly generated 2D metrics. GA is run on each metric with 10 separate random initializations of the population. The red and green traces show the performance of the GA search while black trace shows the performance of StOP. The dashed-line, the solid line, and the dotted line corresponds to the 10th percentile, median, and the 90th percentile of the number of function evaluations for the correspondingly colored algorithm. For proper visualization, the plot is cropped at an upper limit of 200 which is much below the maximum allowed number of evaluations at 10,000.

We compare the performance of *m*-ary search, equivalent to running StOP on a one-parameter one-metric system, to binary search, to illustrate the improved efficiency of the former (Figure 7A). Assuming a nonstochastic, continuous, monotonic function, we calculate the ratio (*r*) of the input domain to the size of the desired range. For a monotonic function, there is only a single continuous desired range of *x*. In the worst case scenario, the search has to continue until the size of the valid feasible range becomes less than or equal to the size of the desired range for *x*. The number of iterations required to reach there can be calculated exactly based on the reduction in the search space per iteration. This is proportional to the time taken, assuming all the *m* candidate points can be explored in parallel, and the IMP runs roughly take the same amount of time. We observe that larger the *r*, and greater the *m*, the greater is the advantage of using *m*-ary search over binary search.

### 3.3. Comparison to Genetic Algorithm Search

We compare the performance of StOP to that of the GA-search by comparing the number of metric function evaluations. In the context of integrative modeling, these evaluations would be IMP runs which are expensive and can significantly increase the runtime of the algorithm, thereby limiting its utility. Within the maximally allowed 10,000 metric evaluations, >90% of the 200 searches with GA converged to a solution. StOP was able to find a solution in all 20 examples. Figure 7B shows the comparison of metric evaluations for StOP and GA at different maximum mutation sizes and different sizes of the population. The median number of metric evaluations for GA with a population of 5 (red solid line) at lower maximum-mutation-sizes is slightly lower than that of StOP (black solid line). However, this is not the case at higher maximum mutation sizes or for GA with a population of 50 (green solid line). More importantly, the 90th percentile (dotted lines) for GA searches is markedly higher than that of StOP at all maximum mutation sizes, which would result in a much greater time needed for convergence. We note that the optimal choice of maximum mutation size and the population size heavily depends on the specific metric used and is not known a priori, rendering the choice of these hyperparameters essentially subjective which may result in suboptimal selections in practice.

### 3.4. IMP Systems

**Figure 8:**
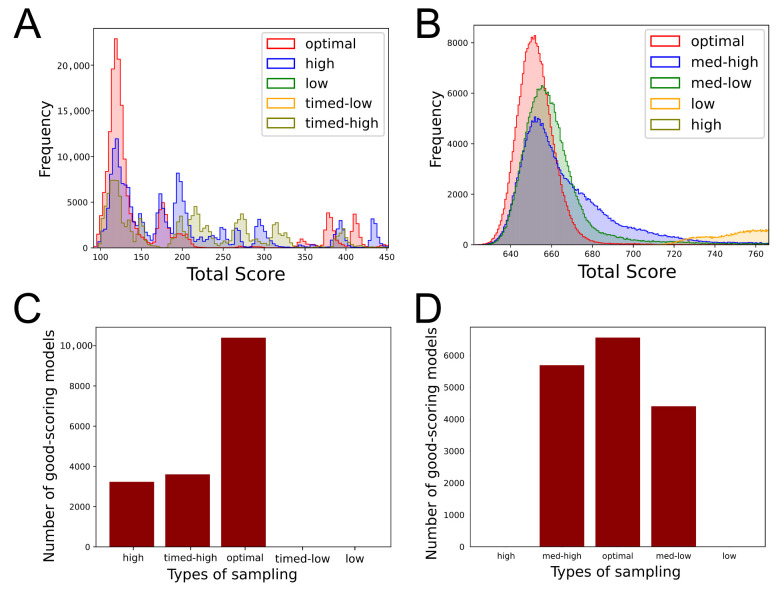
The performance of StOP on integrative modeling of the actin–tropomyosin system [11] and γtusc-Spc110 system [39] systems. (**A**) Histograms showing the total score distribution for all the sampled models for the actin system. For visualization, the upper cutoff of the visible histogram is set at the 99th percentile of the scores for the *optimal* set. (**B**) Histograms showing the total score distribution for all the sampled models for the γtusc system. For visualization, the upper cutoff of the visible histogram is set at the 99.7th percentile of the scores for the *optimal* set. (**C**) Barplot for the number of good-scoring models obtained for the different types of sampling for the actin system. (**D**) Barplot for the number of good-scoring models obtained for the different types of sampling for the γtusc system.

The total score distribution of the sampled models in both the systems indicates that the *optimal* set produced more models with lower scores, i.e., higher posterior probability, as visualized by the higher density toward the left for the *optimal* set compared to the others (Figure 8A,B). For both systems, the total number of models consistent with the input data, i.e., good-scoring models, was higher for the *optimal* set than all other sets (Figure 8C,D). There were no good-scoring models for the *low* set for both the systems, *high* set for the γtusc system, and the *timed-low* set for the actin system. Further, for both systems, the *optimal* set also had a relatively smoother posterior distribution for each restraint in comparison to the other sets (Supplementary Figure S1A–F). Notably, the actin *timed-high* set has a visibly bimodal distribution of the total score of the good-scoring models, which would indicate insufficient sampling. (Supplementary Figure S1D)

For actin, the *optimal*, *high*, and *timed-high* sets produced sampling precisions of 2.375 Å, 3.4 Å, and 2.5 Å, respectively. The *timed-high* set had a similar sampling precision to the optimal set; however, we note that it barely passed the score convergence test (Kolmogorov–Smirnov test *p*-value: <0.001, effect size D: 0.2997) [9] while the rest of the sets showed score convergence. For γtusc, the *optimal*, *med-high*, and *med-low* sets produced sampling precisions of 4.456 Å, 5.156 Å, and 5.062 Å.

For actin, the time taken per model sampled was lowest for the *optimal* set and highest for the *low* set (Supplementary Figure S2A). For γtusc, the times taken per model sampled are similar for all types of sampling (Supplementary Figure S2B). The time difference depends on the specific restraints used and the computational optimizations underlying IMP. In practice, the difference in running time significantly affects the amount of sampling that can be undertaken. For both the systems, the localization densities are visibly similar for all the sets (Supplementary Figure S2C,D).

## 4. Discussion

Here, we list the advantages, disadvantages, uses, and future directions of StOP, simultaneously contrasting it with other optimization methods.

### 4.1. Advantages

StOP converges in a few iterations and makes flexible use of available computing cores, allowing for the optimization of a large number of parameters. The dependence between parameters is explicitly considered during optimization. Finally, it only requires that the metrics be continuous functions. It does not require gradients and step sizes, is insensitive to a single starting point, and does not require additional parameters.

#### 4.1.1. Parallelism

Some methods such as binary [30], interpolation search [31], and golden-section search [32] are sequential, sampling a few new candidate solutions in each iteration, while others can be parallelized to various degrees. For example, the D directions can be evaluated concurrently in a directional search algorithm; the shrink, expansion, and reflection operations can be parallelized in the Nelder–Mead method; and gradients can be estimated by concurrent evaluations of the objective function in stochastic approximation [19]. StOP makes flexible use of available computing cores, achieving two kinds of parallelism. First, all independent parameter groups are optimized in parallel. Second, different feasible regions of a single parameter group are explored in parallel through the m-ary search.

#### 4.1.2. Local versus Global Search

In contrast to local search methods such as Nelder–Mead [20], pattern search [21,22,23,24,25,26], stochastic approximation [27,28], and random search [29], the parallel exploration of multiple feasible regions and the DFS-based branch-and-bound search strategy makes our algorithm a global optimization method. As a result, StOP does not depend on the starting point, gradients, and step sizes and is likely to be faster and more accurate than the local search methods for rugged landscapes. It is similar in spirit to global search methods such as DIRECT [34] and MCS [35], which also partition the search space into progressively smaller spaces along a single-coordinate axis and backtrack to a different feasible region if the search in the current region ends. Backtracking is a particularly useful feature for a multiobjective optimization method since a single feasible region might not always result in a solution that optimizes all metrics (Figure 6B).

#### 4.1.3. Constraints on the Objective Function and Method Parameters

Bayesian optimization requires a prior distribution of the objective function [38]. Stochastic approximation methods require a large number of parameters to estimate the gradients at each step and requires the objective function to be strongly concave/convex [27,28]. By contrast, StOP only requires the objective function to be continuous and does not require any further knowledge of the objective function.

#### 4.1.4. Number of Function Evaluations

Methods that require a large number of function evaluations at each step such as genetic algorithms [36] (see Section 3), particle swarm method [37], and Bayesian optimization [38] are not suitable for our problem. This is because each function evaluation is expensive, as it depends on the output of several IMP MCMC runs, which can in turn be time consuming.

### 4.2. Disadvantages

First, StOP requires some user knowledge. For example, the user needs to know the parameters to be optimized, the parameter-to-metric mapping, the input domain of the parameters, target ranges of the metrics, and the amount of sampling necessary for obtaining sufficient statistics on the metrics. Second, StOP is not efficient on high-dimensional landscapes (high *n*). For such problems, gradient-based methods are likely to be more efficient. Finally, it is a heuristic and not guaranteed to find the target range in complex landscapes. For example, StOP is more likely to fail if the target metric range is near the maxima or minima (Figure 6D).

### 4.3. Uses

First, the use of StOP results in a more efficient sampling of the posterior distribution, resulting in more good-scoring models and higher sampling precision. Equivalently, StOP can result in a more accurate estimation of the structure, and its precision if the available time for sampling is limited. Second, it can be used to make informed choices on modeling criteria, such as the model representation (stoichiometry, number of states, and coarse graining), restraint-related parameters and restraints weights, and the sampling degrees of freedom [42]. Third, the method is flexible in terms of the metrics that can be optimized, and a user can easily implement a metric of their own choosing. Finally, the method is generally adaptable to optimization problems with a target range of the metric, but this is greatly dependent on the landscape. Additionally, we note that StOP could be used in conjunction with gradient-based methods. If the input domain is very large, it can be helpful to run StOP in an initial global search to narrow down the feasible range; following which, sophisticated local search methods such as gradient-based methods could be used.

### 4.4. Future Directions

In an improvement on the current method, one can dynamically vary m(n) and sample the hyper-rectangles bounded by grid points instead of just the grid lines in case of *n*-D search. In addition, sampling would be more efficient if move sizes can be adaptively set instead of having to be optimized in the beginning of a modeling run [17]. Finally, enhancements such as these could eventually lead to complete automation of the integrative modeling protocol, enabling efficient and accurate structure determination of large assemblies on a routine basis.

## Data Availability

All the code for StOP and the scripts necessary to reproduce the paper data and figures is available at https://github.com/isblab/stop (accessed on 20 October 2021).

## Supplementary Materials

The following are available at https://www.mdpi.com/article/10.3390/life11111183/s1, Figure S1: Score distributions for the different restraints in Actin and γtusc systems, Figure S2: The time-efficiency of the different types of sampling and the localization densities, Table S1: The performance of StOP on random metrics, Supplementary Section S1: Analysis Details, Supplementary Section S2: Default value of *m*(*n*), Supplementary Section S3: Random Functions, Supplementary Section S4: Platform and Packages.

## References

[1] Alber F., Dokudovskaya S., Veenhoff L.M., Zhang W., Kipper J., Devos D., Suprapto A., Karni-Schmidt O., Williams R., Chait B.T. (2007). Determining the architectures of macromolecular assemblies. Nature.

[2] Ward A.B., Sali A., Wilson I.A. (2013). Integrative Structural Biology. Science.

[3] Rout M.P., Sali A. (2019). Principles for Integrative Structural Biology Studies. Cell.

[4] Yu Y., Li S., Ser Z., Sanyal T., Choi K., Wan B., Kuang H., Sali A., Kentsis A., Patel D.J. (2021). Integrative analysis reveals unique structural and functional features of the Smc5/6 complex. Proc. Natl. Acad. Sci. USA.

[5] Ganesan S.J., Feyder M.J., Chemmama I.E., Fang F., Rout M.P., Chait B.T., Shi Y., Munson M., Sali A. (2020). Integrative structure and function of the yeast exocyst complex. Protein Sci..

[6] Gutierrez C., Chemmama I.E., Mao H., Yu C., Echeverria I., Block S.A., Rychnovsky S.D., Zheng N., Sali A., Huang L. (2020). Structural dynamics of the human COP9 signalosome revealed by cross-linking mass spectrometry and integrative modeling. Proc. Natl. Acad. Sci. USA.

[7] Kim S.J., Fernandez-Martinez J., Nudelman I., Shi Y., Zhang W., Raveh B., Herricks T., Slaughter B.D., Hogan J.A., Upla P. (2018). Integrative structure and functional anatomy of a nuclear pore complex. Nature.

[8] Viswanath S., Bonomi M., Kim S.J., Klenchin V.A., Taylor K.C., Yabut K.C., Umbreit N.T., Van Epps H.A., Meehl J., Jones M.H. (2017). The molecular architecture of the yeast spindle pole body core determined by Bayesian integrative modeling. Mol. Biol. Cell.

[9] Viswanath S., Chemmama I.E., Cimermancic P., Sali A. (2017). Assessing Exhaustiveness of Stochastic Sampling for Integrative Modeling of Macromolecular Structures. Biophys. J..

[10] Webb B., Viswanath S., Bonomi M., Pellarin R., Greenberg C.H., Saltzberg D., Sali A. (2018). Integrative structure modeling with the Integrative Modeling Platform: Integrative Structure Modeling with IMP. Protein Sci..

[11] Saltzberg D., Greenberg C.H., Viswanath S., Chemmama I., Webb B., Pellarin R., Echeverria I., Sali A., Bonomi M., Camilloni C. (2019). Modeling Biological Complexes Using Integrative Modeling Platform. Biomolecular Simulations.

[12] Saltzberg D.J., Viswanath S., Echeverria I., Chemmama I.E., Webb B., Sali A. (2021). Using Integrative Modeling Platform to compute, validate, and archive a model of a protein complex structure. Protein Sci..

[13] Russel D., Lasker K., Webb B., Velázquez-Muriel J., Tjioe E., Schneidman-Duhovny D., Peterson B., Sali A. (2012). Putting the Pieces Together: Integrative Modeling Platform Software for Structure Determination of Macromolecular Assemblies. PLoS Biol..

[14] Rieping W. (2005). Inferential Structure Determination. Science.

[15] Roberts G.O., Rosenthal J.S. (2001). Optimal scaling for various Metropolis-Hastings algorithms. Stat. Sci..

[16] Rosenthal S. (2009). Optimal Proposal Distributions and Adaptive MCMC. Handbook of Markov Chain Monte Carlo.

[17] Roberts G.O., Rosenthal J.S. (2007). Coupling and Ergodicity of Adaptive Markov Chain Monte Carlo Algorithms. J. Appl. Probab..

[18] Di Pierro M., Elber R. (2013). Automated Optimization of Potential Parameters. J. Chem. Theory Comput..

[19] Larson J., Menickelly M., Wild S.M. (2019). Derivative-free optimization methods. Acta Numer..

[20] Nelder J.A., Mead R. (1965). A Simplex Method for Function Minimization. Comput. J..

[21] Fermi E., Metropolis N. (1952). Numerical Solution of a Minimum Problem.

[22] Hooke R., Jeeves T.A. (1961). “ Direct Search” Solution of Numerical and Statistical Problems. J. ACM.

[23] Rosenbrock H.H. (1960). An Automatic Method for Finding the Greatest or Least Value of a Function. Comput. J..

[24] Torczon V. (1991). On the Convergence of the Multidirectional Search Algorithm. SIAM J. Optim..

[25] Booker A.J., Dennis J.E., Frank P.D., Serafini D.B., Torczon V., Trosset M.W. (1999). A rigorous framework for optimization of expensive functions by surrogates. Struct. Optim..

[26] Audet C., Dennis J.E. (2006). Mesh Adaptive Direct Search Algorithms for Constrained Optimization. SIAM J. Optim..

[27] Robbins H., Monro S. (1951). A Stochastic Approximation Method. Ann. Math. Stat..

[28] Kiefer J., Wolfowitz J. (1952). Stochastic Estimation of the Maximum of a Regression Function. Ann. Math. Stat..

[29] Rastrigrin L. (1963). The Convergence of the Random Search Method in the External Control of Many-Parameter System. Autom. Remote Control..

[30] Cormen T.H., Leiserson C.E., Rivest R.L., Stein C., Cormen T.H. (2009). Introduction to Algorithms.

[31] Peterson W.W. (1957). Addressing for Random-Access Storage. IBM J. Res. Dev..

[32] Kiefer J. (1953). Sequential minimax search for a maximum. Proc. Am. Math. Soc..

[33] Shubert B.O. (1972). A Sequential Method Seeking the Global Maximum of a Function. SIAM J. Numer. Anal..

[34] Jones D.R., Perttunen C.D., Stuckman B.E. (1993). Lipschitzian optimization without the Lipschitz constant. J. Optim. Theory Appl..

[35] Huyer W., Neumaier A. (1999). Global Optimization by Multilevel Coordinate Search. J. Glob. Optim..

[36] Holland J.H. (1962). Outline for a Logical Theory of Adaptive Systems. J. ACM.

[37] Kennedy J., Eberhart R. (1995). Particle swarm optimization. Proc. Int. Conf. Neural Netw..

[38] Mockus J. (1994). Application of Bayesian approach to numerical methods of global and stochastic optimization. J. Glob. Optim..

[39] Brilot A.F., Lyon A.S., Zelter A., Viswanath S., Maxwell A., MacCoss M.J., Muller E.G., Sali A., Davis T.N., Agard D.A. (2021). CM1-driven assembly and activation of yeast *γ*-tubulin small complex underlies microtubule nucleation. eLife.

[40] Tange O. GNU Parallel 20200622 (’Privacy Shield’); Zenodo. https://zenodo.org/record/3956817#.YYEFRJpByUk.

[41] Pettersen E.F., Goddard T.D., Huang C.C., Couch G.S., Greenblatt D.M., Meng E.C., Ferrin T.E. (2004). UCSF Chimera: A visualization system for exploratory research and analysis. J. Comput. Chem..

[42] Viswanath S., Sali A. (2019). Optimizing model representation for integrative structure determination of macromolecular assemblies. Proc. Natl. Acad. Sci. USA.

