# Supplementary material for: A Framework for Stochastic Optimization of Parameters for Integrative Modeling of Macromolecular Assemblies
Source: Life (Basel). 2021 Nov 5;11(11):1183. doi: 10.3390/life11111183 (PMC8618978; doi:10.3390/life11111183)
Supplement: Supplementary file 1 [file life-11-01183-s001.zip › life-1411967-supplementary.pdf]

Supplementary for **A Framework for Stochastic Optimization of Parameters for Integrative Modeling of Macromolecular Assemblies**

Satwik Pasani[1] and Shruthi Viswanath[1]

[1]National Center for Biological Sciences, Tata Institute of Fundamental Research, Bangalore, India
*Correspondence: shruthiv@ncbs.res.in

1 Analysis Details

First, the runs are tested for equilibration. The mean of the final quarter of the frames is compared to that of the preceding quarter. The algorithm warns that the run is not equilibrated if either mean is outside of 2 standard deviations of the other mean. Otherwise, the second half of each run is considered equilibrated and used for further analysis.

Next, by default, metric values for each equilibrated frame are extracted based on the match between the user-specified search string for a metric and the IMP stat file fields. For Monte Carlo acceptances, including Replica Exchange swap success ratios, PMI outputs cumulative acceptances and this is adjusted to obtain non-cumulative acceptances. Finally, metric values are averaged across all matching fields in a single frame, across the different temperature replicas if applicable, across all the equilibrated frames of a run, and finally across all the replicate runs. Further, a user also has the option of implementing their own metrics or the analysis scripts.

2 Default value of $m(n)$

The default value of the $m(n)$ function is calculated as follows. The CPU count for the system is calculated and the number of replicates is set to the default value of 3 (unless overridden by the user). $m(1)$ is set to $\lfloor \text{no of CPU/replicates} \rfloor$ which allows us to run all the replicates of a 1D search as a single parallel block. We ensure that $m(n)$ is at least 3, setting it to

$$m(n) = \max(3, \lfloor (s \times m(1))^{1/n} \rfloor)$$

which allows the root node of a parameter group with n parameters to finish in s blocks if $(s \times m(1))^{1/n}$ is an integer. The algorithm currently just sets s to 3.

3 Random Functions

To generate an unbiased variety of example 1D landscapes, the following strategy was used. For all the example cases, we fix the target range to be $[0.48, 0.5]$. We first generate h uniformly distributed pseudorandom numbers in $[0, 1]$. We then consider these to be the values of the random metric at h linearly spaced points between $[-1, 1]$. To ensure that there is a desired range of the parameter between $[-1, 1]$, we scale the obtained values such that the minimum of the scaled values lies in $[-1, 0.4]$ and the maximum lies in $[0.6, 2]$. The complete function is calculated by using cubic interpolation on these values. For the 2D random metrics, we repeat the same procedure as above, but we begin by creating h^2 random points in 2D and then use Rectilinear Cubic

Splines without smoothing to interpolate. The random functions were generated by varying from 5 to 25 in increments of 5. At each fixed h, we generate 20 different random functions. StOP is run on each of these functions with $m(1) = 3, 5$ and 7 for 1D examples and $m(2) = 3, 5$ and $m(1) = 5$ for the 2D examples. All the generated functions with the illustration of StOP on them (in accordance with the scheme in Figure 5) are available at https://github.com/isblab/stop/. Here, h roughly determines the ruggedness of the function since it controls how undulating the landscape can be. For higher h, increasing m increases the chance of success. Maximum DFS depth was capped at 4 for all the optimizations.

4 Platform and Packages

All the analysis was done with python 3.8 (matplotlib 3.4.1, scipy 1.6.2, numpy 1.21.2). IMP version used was 2.14. GNU Parallel was used to run the IMP runs and UCSF Chimera was used for the visualization of the densities in Supplementary Figure S2. Other libraries used in the code are listed on GitHub https://github.com/isblab/stop.

5 Tables and Figures

n, m	$h = 5$	$h = 10$	$h = 15$	$h = 20$	$h = 25$
1-D, $m(1) = 3$	17	14	10	12	12
1-D, $m(1) = 5$	20	17	15	16	18
1-D, $m(1) = 7$	20	20	18	19	18
2-D, $m(2) = 3, m(1) = 5$	18	20	20	19	18
2-D, $m(2) = m(1) = 5$	20	20	20	20	20

Table S1: **The performance of StOP on random metrics.** The columns represent the different values of h which roughly controls the level of ruggedness of the random function by controlling the number of random points generated for interpolation (Supplementary Section S3). Rows represent varying values of $m(n)$ for StOP. The values in the cell represent the number of examples out of a total of 20 examples for which StOP was able to successfully find a solution.

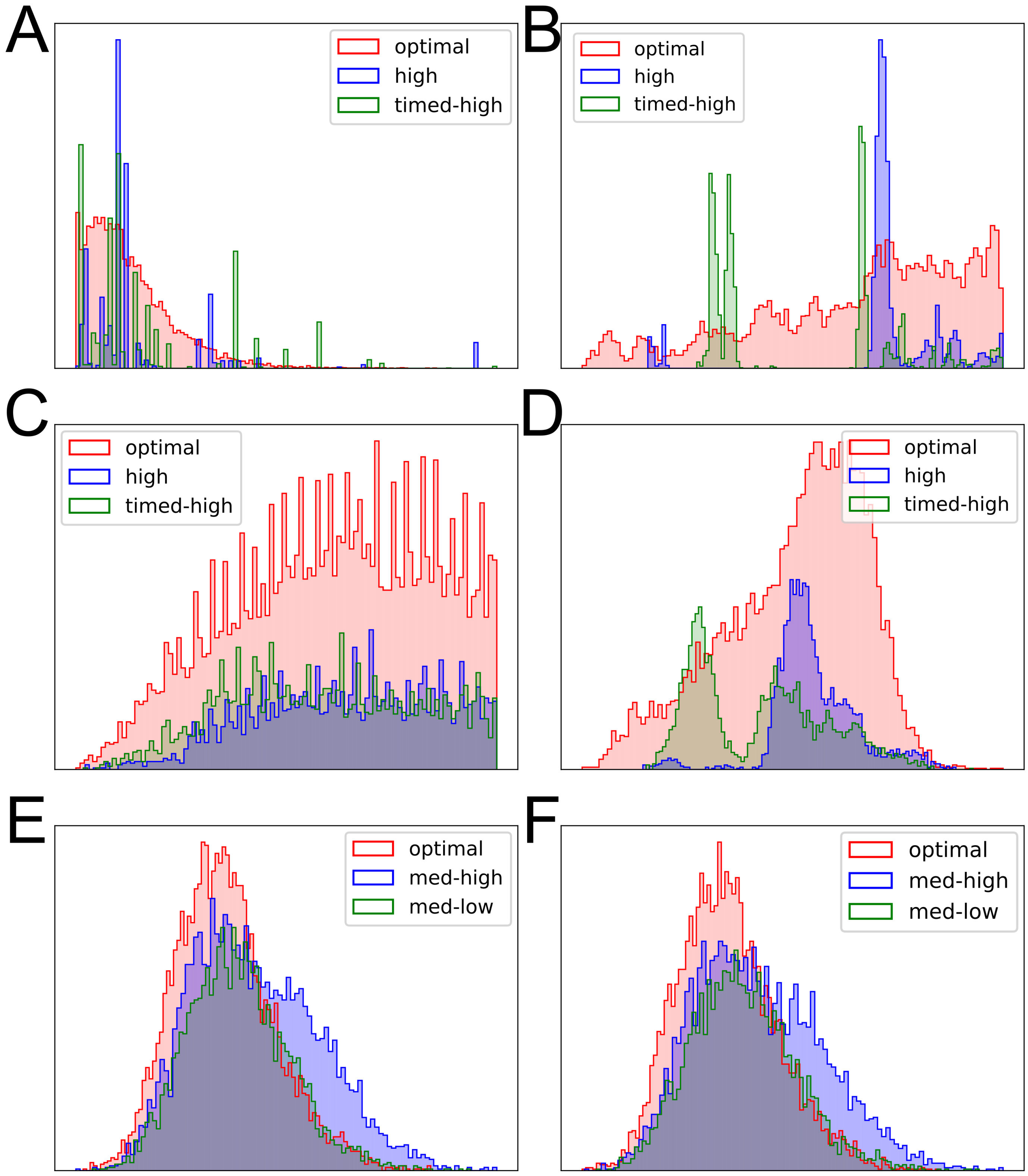

Figure S1: **Score distributions for the different restraints in Actin and γtusc systems. A)** Cross Linking restraint score for the actin system. **B)** EM-restraint score for the actin system. **C)** SAXS restraint score for the actin system. **D)** Total score for the actin system. **E)** Cross Linking restraint score for the γtusc system. **F)** Total score for the γtusc system. All the histograms represents only the good-scoring models. The x-axis of all panels represents the restraint score, and the y-axis represents the frequency.

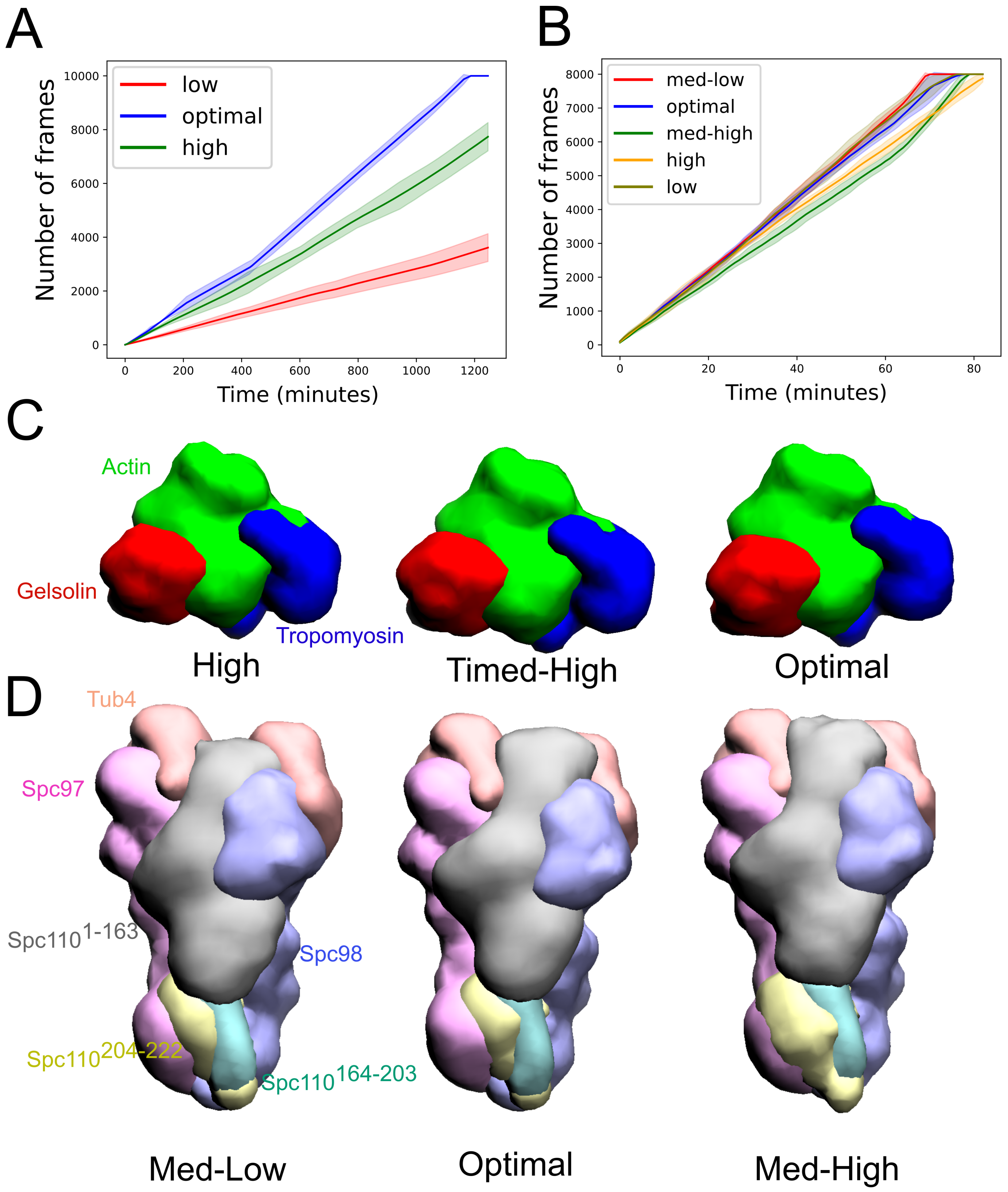

Figure S2: **The time-efficiency of the different types of sampling and the localization densities. A)** Time taken vs the number of frames (sampled models) for the actin system. **B)** Time taken vs the number of frames for the γtusc system. **C)** Localization densities of the largest cluster of models for the actin system visualized using UCSF Chimera. The threshold for density visualization is set to 0.05 D) Localization densities of the largest cluster of models for the γtusc system. The threshold for density visualization is set to 1 SD. The domains representing the densities are labelled in the first figure with corresponding colors for panels C and D.
